# Estimating a Seismic Wave Velocity for Exciting the Greatest Anticipated Vertical Deck Displacement of a Cable-Stayed Bridge Subjected to Asynchronous Excitation

**DOI:** 10.1186/s40069-020-00450-9

**Published:** 2021-02-04

**Authors:** Bashar Hariri, Lan Lin

**Affiliations:** 1 Department of Building, Civil and Environmental Engineering, Concordia University, Montreal, QC H3G 1M8 Canada; 2 Department of Building, Civil and Environmental Engineering, Concordia University, 1455 de Maisonneuve Blvd, W., EV006.245, Montreal, QC H3G 1M8 Canada

**Keywords:** cable-stayed bridges, vertical displacement, deck, asynchronous excitation, resonance, period, wave velocity, time delay

## Abstract

The purpose of this study is to examine the effects of the seismic wave velocity on vertical displacement of a cable-stayed bridge’s deck under asynchronous excitation. The Quincy Bayview Bridge located in Illinois, USA, and four other generic bridges are selected for the study. Ten records obtained from earthquakes in US, Japan, and Taiwan are used as input for the seismic excitation in the time-history analysis. Two equations are proposed in this study to determine a critical seismic wave velocity that would produce the greatest vertical deck displacement. The critical wave velocity depends on the total length of the bridge, the fundamental period of the bridge, and the *C-factor*. The *C-factor* in this study is 0.72, which is based on analyzed results from the five selected bridges. The two equations and the *C-factor* are verified through application on two 3-span cable-stayed bridges studied previously by Nazmy and Abdel-Ghaffar. The proposed C-factor of 0.72 is recommended for use for typical 3-span cable-stayed bridges with a side-to-main span ratio of about 0.48. The methodology developed in the study, however, can be applied to any specific bridge to examine the excitation of the deck vertical displacement under the longitudinal seismic ground motion.

## Introduction

Records show that long-span bridges, such as cable-stayed bridges, are vulnerable to earthquakes. Some notable examples are: the M_w_ 5.9 earthquake that occurred in 1988 in Saguenay, Quebec, Canada, caused a failure of one anchorage plate in the 183-m-long Shipshaw Bridge located about 40 km from the epicenter (Filiatrault et al. 1993); the 3-span cable-stayed Higashi-Kobe Bridge was severely damaged during the 1995 M_w_ 6.9 Kobe earthquake, damage of the connections and wind shoe, as well as buckling of cross beams and pier leg were observed at the west pier of the bridge (Wilson 2003); due to the 1999 M_w_ 7.7 Chi-Chi earthquake, damage to the girder, the cable, the pylon and the pier in the 2-span (199.9 m + 199.9 m) Ji-Ji-Da Bridge occurred (Kosa and Tasaki 2003); and in the same Chi-Chi earthquake, the shear keys, the pylon and one cable were damaged in the 2-span cable-stayed Chi-Lu Bridge (Chang et al. 2004).

Extensive research has been conducted over the last 30 years in order to find ways of protecting cable-stayed bridges from damage caused by earthquakes. One of the major discoveries has been that asynchronous excitation should be used as input in the time-history analysis of cable-stayed bridges. As the bridge supports are largely separated in cable-stayed bridges, the lack of the synchronism of the ground motion between the bridge supports is due to the wave passage effect, the incoherence effect, and the local site effect. It has been confirmed in several studies that the incoherence effect is less important than the wave passage effect and can be ignored in the seismic analysis of cable-stayed bridges (Abdel-Ghaffar 1991; Priestley et al. 1996; De Silva 2005; Camara 2018). Furthermore, it has been recognized that the overall load–displacement relationship for cable-stayed bridges is nonlinear under design or service loads as reported in Fleming (1979); Nazmy and Abdel-Ghaffar (1990a). The geometric nonlinearity originates from the sag effect of the inclined cables due to their own weight, the nonlinear behavior of bending members, and the geometry changes caused by large displacements. On the other hand, Fleming (1979), Fleming and Egeseli (1980), and Nazmy and Abdel-Ghaffar (1990b) concluded that geometric nonlinearity had a minor effect on the seismic behavior of cable-stayed bridges. Accordingly, the small deformation method in conjunction with material nonlinearity is adequate for the seismic analysis of this kind of bridges.

Despite the numerous related studies, most modern design codes or standards do not provide detailed guidelines for the seismic analysis of cable-stayed bridges under asynchronous excitation. These codes include the AASHTO Guide Specifications for LRFD Seismic Bridge Design (AASHTO 2015), the Canadian Highway Bridge Design Code (CSA 2014), and the Japanese Design Specifications Highway Bridges (PWRI 1998). The only seismic code presenting a step-by-step procedure to account for the asynchronous excitation in the analysis of bridges for design purpose is the Eurocode 8 (CEN 2004). Although the EC8 approach is simple and straight forward, specific drawbacks are that it ignores the higher mode effects and the characteristics of the records selected for the time-history analysis (Sextos and Kappos 2005; Crewe and Norman 2006).

Some researchers have performed seismic analysis on existing cable-stayed bridges to understand their response and performance. Ren and Obata (1999) conducted a study on a 4-span cable-stayed bridge with a central span of 650 m. The purpose of the study was to evaluate the effect of geometric and material nonlinearity on the seismic behavior of the bridge. Four cases were considered in the analysis, namely, small deformation and linear elastic material, geometric nonlinearity and linear elastic material, small deformation and elastic–plastic material, and geometric nonlinearity and elastic–plastic material. The results of this study showed that the material remains elastic under the considered excitation. The study also confirmed that geometric nonlinearity had a very minor effect on the bridge response as reported in previous studies. Liu et al. (2014) investigated response of a 3-span, 576-m-long bridge located in China using three earthquake records. Material nonlinearity was only considered for piers whose behavior was assumed to follow an elastic–plastic hysteretic model proposed by Clough and Johnston (1966). Seismic excitation was applied along the longitudinal direction of the bridge. The study reported that the bridge longitudinal direction was weak, as a result, the longitudinal displacement of piers yielded and the piers underwent significant residual deformation. Siringoringo et al. (2014) examined the performance of the 860-m-long Yokohama Bridge, the second longest cable-stayed bridge in eastern Japan, under the 2011 M_w_ 9.0 earthquake. It was found that the movement of the bridge in the transverse direction dominated the response of both girders and piers, and that there was no damage to the bridge since the amplitude of the earthquake was below that for which the bridge had been designed. Naderian et al. (2016) developed an integrated finite strip method for the seismic analysis of long-span bridges. The KSM cable-stayed bridge located in Hong Kong, which has 3 spans (160 m + 430 m + 160 m), was selected for their study. The vertical displacement at the middle of the main span and the longitudinal displacement at the top of a pylon were recorded to validate the proposed strip method. Zhong et al. (2017) have been one of few researchers to analyze the fragility of cable-stayed bridges under asynchronous excitation. The bridge considered in the study comprised three spans, of which the two side spans were 156 m and the main span was 430 m. One of the main conclusions from their study was that the spatial variability of the ground motion did not affect the fragility curves for the cable, the bearing displacement, or the abutments. In addition to numerical analysis, shaking table tests were carried out to better understand the dynamic behavior of cable-stayed bridges (e.g., Johnson et al. 2008; Cruz Norhuez and Saiidi 2011; Garevski and Severn 1993). Very recently, Zhou et al. (2019) introduced a transverse steel damper system to protect substructures and towers. The proposed system was then tested on a 1:35 model of the 1088-m-long Sutong Bridge built in China. They concluded that the new system presented reliable seismic performance. Guan et al. (2019) also conducted a shake-table test of the Sutong Bridge and reported that the dominant periods obtained from the experiment were consistent with OpenSees results.

## Motivation for the Study

Most of the response parameters selected for examining the performance of the cable-stayed bridges in the aforementioned studies, and other similar studies (e.g., Crewe and Norman 2006; Aswathy et al. 2013; Gong et al. 2015; Shiravand and Parvanehro 2019) are associated with the bridge’s horizontal direction including girder displacement, pylon displacement, and girder moment. Few studies have focused on the vertical displacement of a cable-stayed bridge’s deck. The reason why the response of the bridge deck’s vertical direction is often ignored might be due to the fact that, according to Allam and Datta (2004), the vertical component of the ground motion is always minor. However, to the contrary, Allam and Datta (2004) found that the longitudinal component of the ground motion significantly affected deck vibration in the vertical direction, although the effect was not quantified in their study. Finally, Tian and Lou (2014) claimed that “the structural seismic response may reach an extreme value, or even maxima and minima, at specific apparent wave velocities because of the travelling wave resonance”.

It is interesting to report herein that a few shake-table tests of cable-stayed bridges subjected to non-uniform excitation have been conducted. For example, Yang and Cheung (2011) performed a test on a 1:120 model of a 750-m-long bridge in Hong Kong. Four wave passage velocities, i.e., 400, 600, 1000, and 2000 m/s were considered to determine the difference of the arrival time between the piers. The excitation was applied to the bridge longitudinal direction. The major observation from the study was that wave propagation had a larger effect on the girders than on the towers. Xie et al. (2020) conducted a shake-table test of a bridge with a main span of 1400 m scaled down to 1:70 in order to verify the travelling wave resonance in the seismic response. The seismic excitation was represented by two specific sine waves and applied to the bridge longitudinal direction. Longitudinal displacement at the tops of the towers and tower-girder intersects was monitored in the analysis. It was found in the study that resonance occurred between the travelling wave and the mode.

As discussed above, both numerical analyses (e.g., Allam and Datta 2004; Tian and Lou 2014) and experimental tests (e.g., Xie et al. 2020) have indicated that the vertical displacement of the girder would be excited under the longitudinal seismic ground motion. However, no thorough investigation has been done. This study therefore fills a gap in the literature on this subject. Specifically, the current study examines how the deck vertical displacement changes with the seismic wave velocity where the wave travels along the bridge longitudinal direction, and determines the critical wave velocity that will trigger the maximum deck displacement in the vertical direction. It is noteworthy that determination of the maximum vertical deck displacement of the Bayview Bridge is not within the scope of the study.

## Description and Modeling of Bridge

### Bridge Description

The Quincy Bayview Bridge located in Illinois, USA, was selected for examination as it had been used by several studies to evaluate the performance of cable-stayed bridges (e.g., Hua and Wang 1996; Zadeh 2012; Poddar and Rahman 2015). In addition, the bridge geometry information and ambient vibration test results on the bridge have been well documented in Wilson and Gravelle (1991) and Wilson and Liu (1991), which are essential for developing and validating this study’s finite element model.

**Fig. 1:**
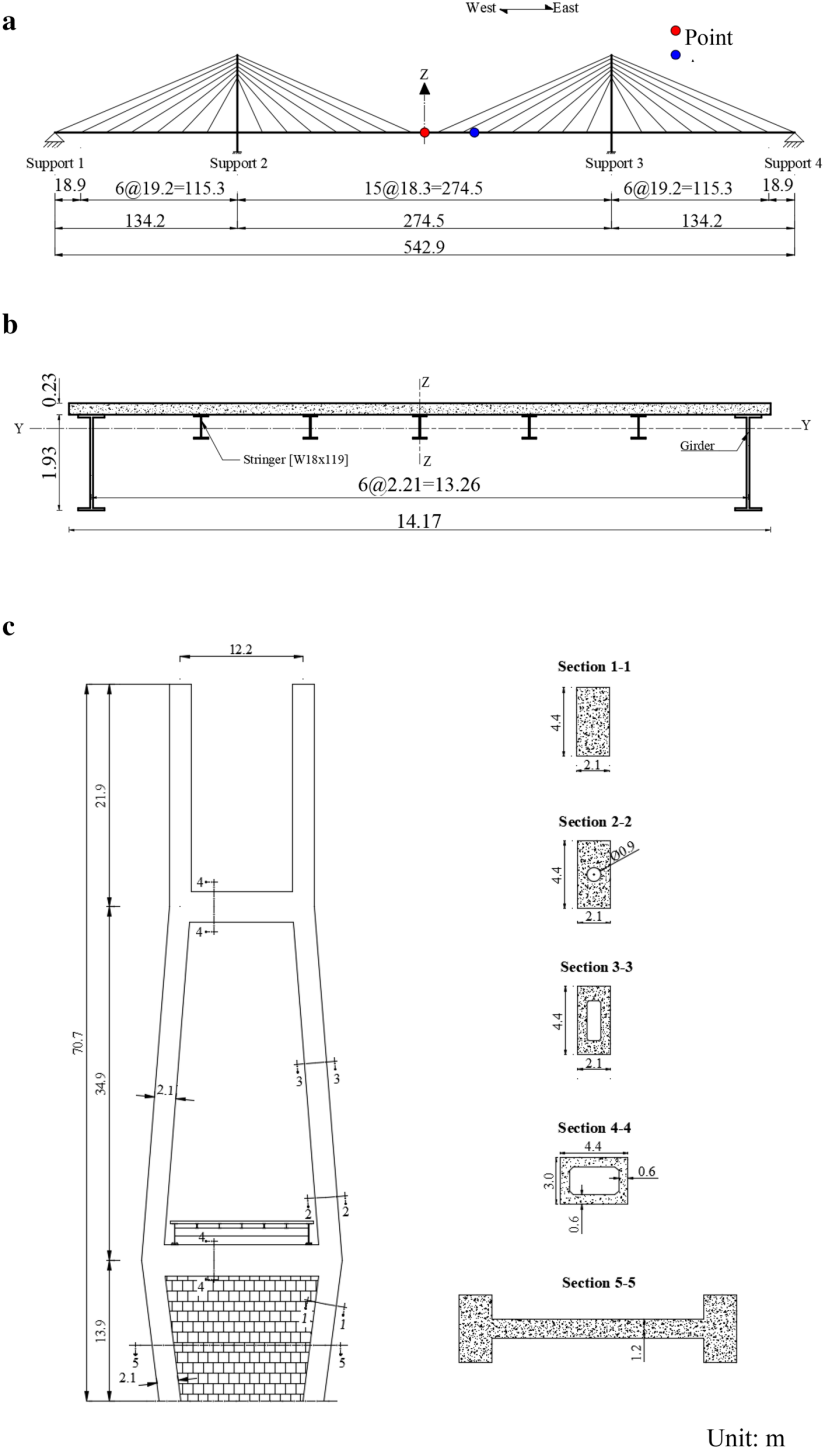
Bridge configuration: **a** elevation view, **b** deck cross section, **c** pylon geometry.

As illustrated in Fig. 1, the Bayview Bridge has three spans of 134.2  + 274.5  + 134.2 m with a total length of 542.9 m. The deck is 0.23 m thick, 14.17 m wide and is made of precast post-tensioned concrete. It is supported by five steel stringers (W18 × 119) equally positioned at a spacing of 2.21 m (center to center). The two main girders at the outer edges of the deck have an overall depth of 1.93 m. Each side span is supported by 14 cables and the main span is supported by 28 cables. All the cables are equally spaced.

The cross section of each tower leg consists of three sections, Section 1-1, Section 2-2, and Section 3-3 (Fig. 1) where Section 1-1 runs from the base of the leg to the level of the deck, Section 2-2 extends about 4.7 m above the deck level, and Section 3-3 runs along the rest of the tower. As shown in Fig. 1, the lower strut supports the entire superstructure and the upper strut connects the two legs at about 48.8 m measured from the bottom of the leg. A 1.2m-thick concrete wall is provided to fill the space between the two legs below the lower strut in order to stiffen the tower.

The superstructure is connected to the towers through two sets of the vertical and transverse bearings at each tower. In addition, longitudinal bearings are installed on the tower at the west end. A tie-down link is used at each bridge end to connect the girder with the abutment.

### Bridge Modeling

**Fig. 2:**
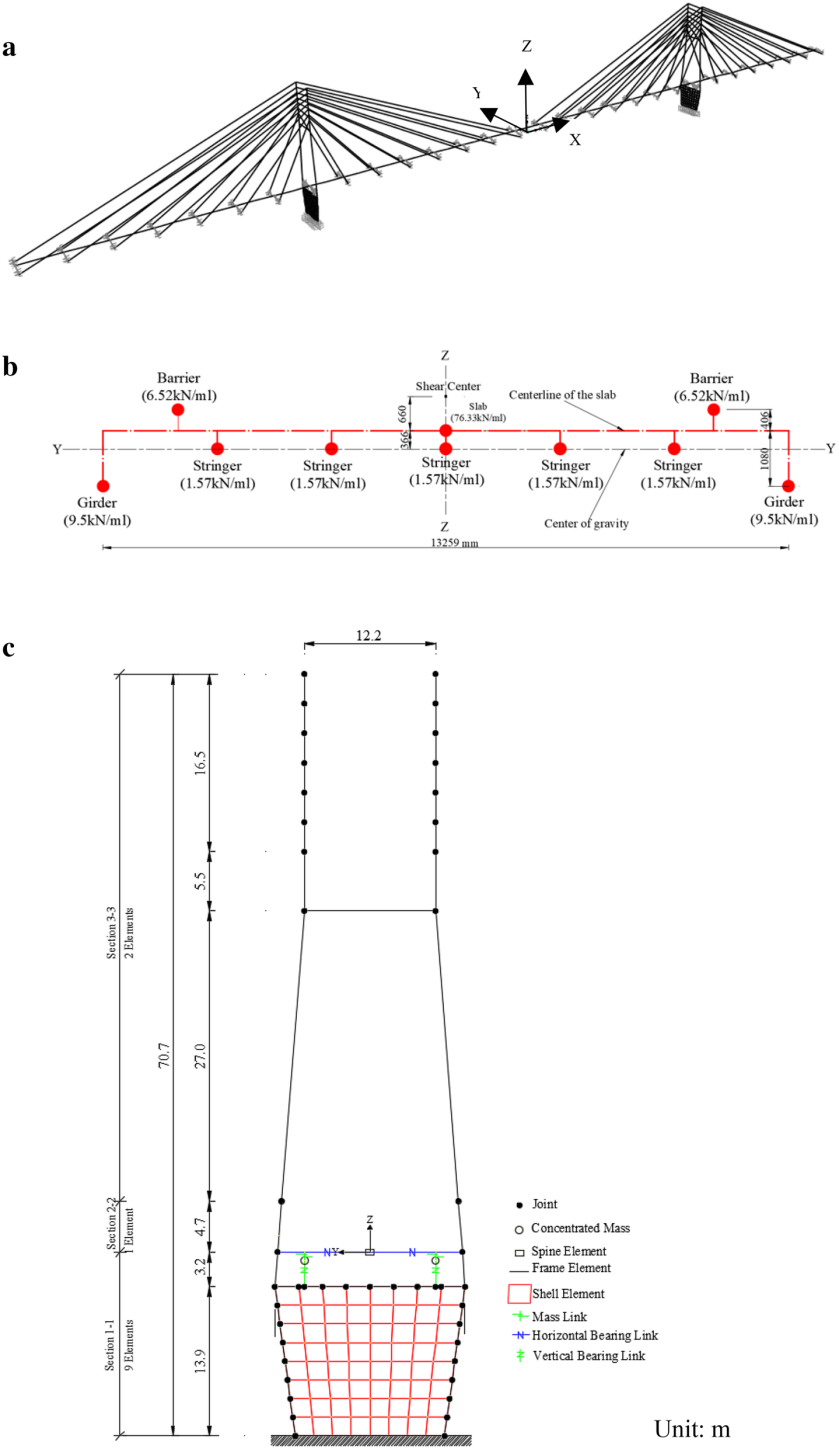
Finite element model of the bridge: **a** 3D spine model, **b** deck, **c** pylon.

Structural analysis software SAP2000 is used to develop a 3D finite element model of the Bayview Bridge (Fig. 2a). Each element on the bridge is modeled following the techniques described in Wilson and Gravelle (1991). A brief summary of the bridge model is presented as follows,

- The superstructure is modeled using 29 spine elements in the bridge longitudinal direction. Figure 2b illustrates the details of the deck model. The weight of each mass and the length of the vertical links are determined by considering the lumped mass and the center of the gravity of each component, including the slab, the girders/stringers, and the barriers.
- The towers are modeled using linear elastic frame elements as illustrated in Fig. 2c. Three types of cross section are defined and assigned to each leg in accordance with the tower geometry presented in Fig. 1. The solid concrete wall below the lower strut is modeled as a shell element and is meshed into 8 × 8 sub-elements.
- Each cable is modeled as a straight frame object (cable) defined in SAP2000.
- The vertical bearings at both towers restrain the movement in the vertical direction. The horizontal bearings only allow the rotation around the axis perpendicular to the bridge longitudinal direction, whereas all three translational degrees of freedom about the x, y, and z axes are fixed.
- The pier bases are assumed to be fully fixed.

It is necessary to mention herein that the input parameters used for developing the model in this study are the same as those reported in Wilson and Gravelle (1991) except the following two parameters, which are based on the latest data published in the literature.

- Damping: Pridham and Wilson (2005) evaluated the damping of the Bayview Bridge based on extensive data collected during ambient vibration tests conducted in 1987. They observed that the damping of the first vertical mode was about 1.4%, and the first transverse-torsional mode was about 1.1%. In addition, they concluded that the damping with a mean of 1.0% and standard deviation of 0.8% was appropriate to assign to all the modes. Given this, a damping of 1.4% was assigned to the first vertical mode and 1.1% was assigned to other modes in the modal analysis. It should be noted that Wilson and Gravelle did not report the value of the damping in their finite element model.
- Modeling of cables: Hua and Wang (1996) reported that using a modified modulus of elasticity of the cable only produced a 2% difference on modal frequencies compared to that without considering cable nonlinearity. Furthermore, they concluded that nonlinear effects on cables could be ignored in the analysis of the Bayview Bridge. Accordingly, each cable in the current study is modeled as one linear segment without sag. On the contrary, Wilson and Gravelle used a single truss element to model each cable in their study.

### Modal Validation

In order to validate the finite element model developed in this study, the dynamic characteristics of the bridge model including mode shapes and modal frequencies from the current study are compared with the analysis results reported in Wilson and Gravelle (1991) and the ambient vibration test results described in Wilson and Liu (1991). For ease of discussion, the results from the current study, the Wilson and Gravelle study, and the Wilson and Liu study are designated as CFEM (current finite element model), Wilson FEM, and Wilson test, respectively.

**Fig. 3:**
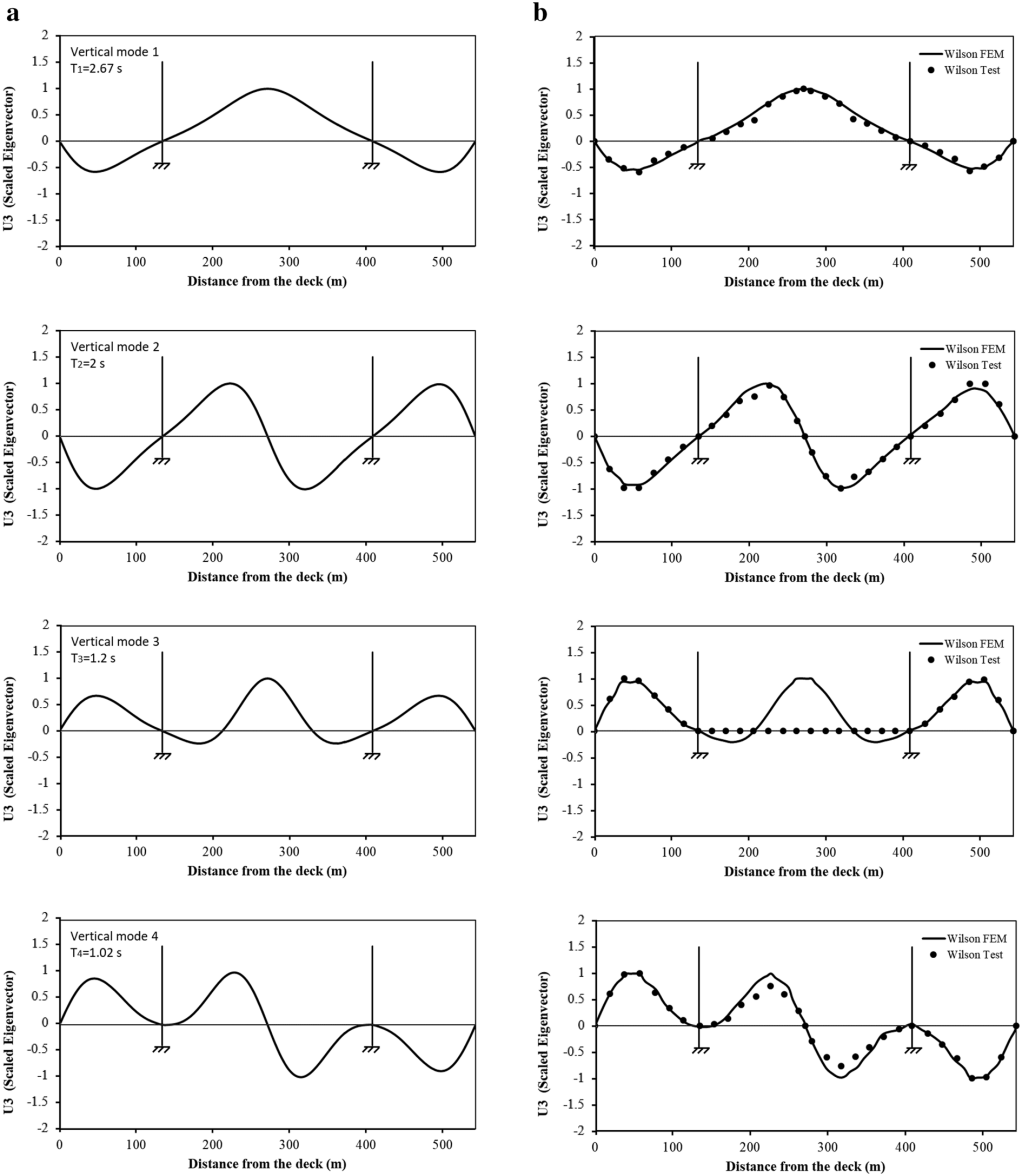
Vertical mode shapes: **a** CFEM results, **b** Wilson FEM and Wilson test results.

**Fig. 4:**
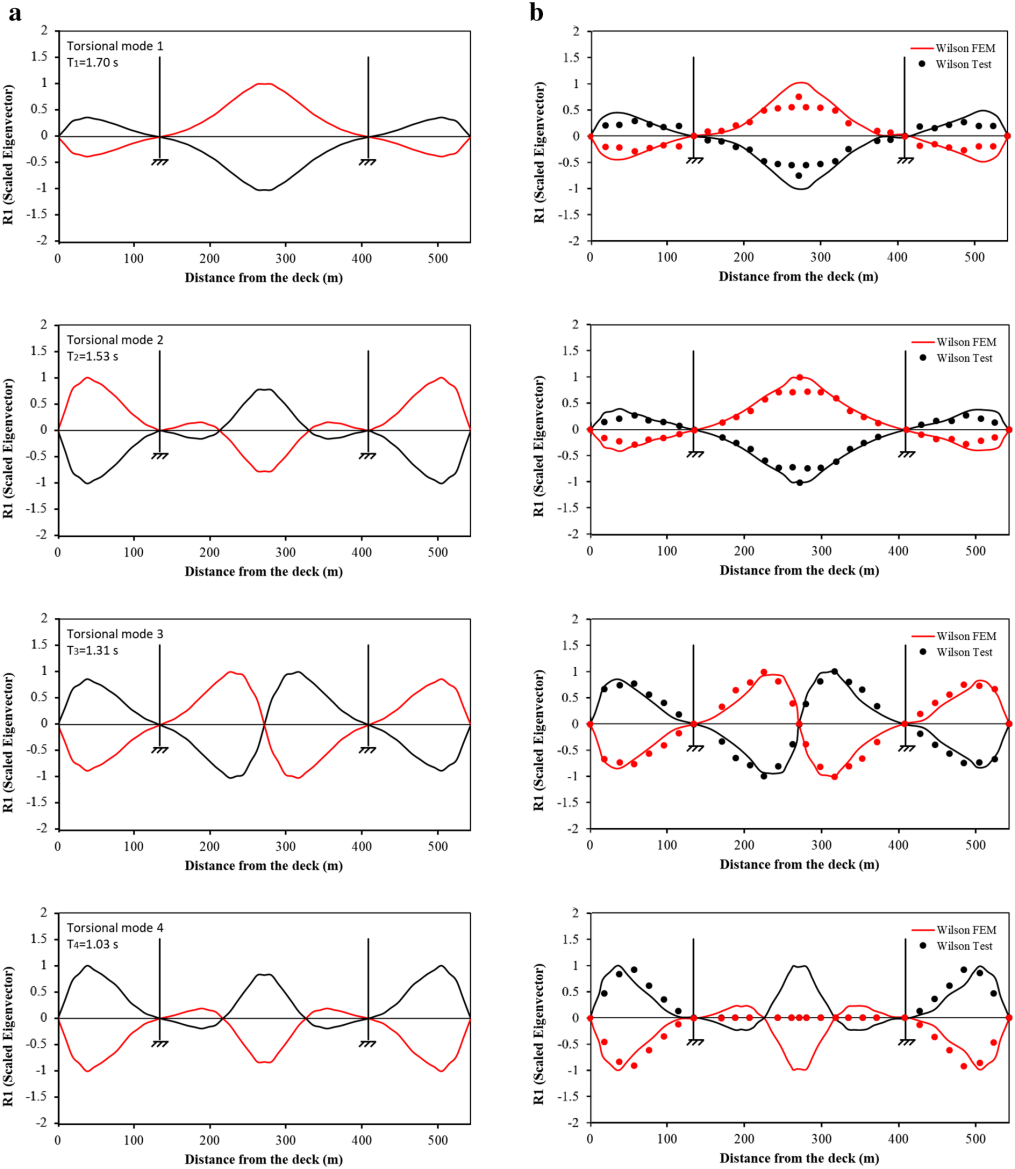
Torsional mode shapes: **a** CFEM results, **b** Wilson FEM and Wilson test results.

Figure 3 presents a comparison of the first four mode shapes for vibration in the vertical direction and Fig. 4 for the torsional modes. It can be seen clearly in Fig. 3 that the CFEM mode shapes are almost identical to Wilson FEM and Wilson test. Such results are not surprising given the fact that the CFEM is developed following the procedure for Wilson FEM. Regarding the torsional modes (Fig. 4), CFEM and Wilson FEM provide almost the same mode shapes except the 2nd mode in which the CFEM Eigen values for the side spans are almost two times the values of Wilson FEM. Furthermore, the CFEM mode shape for the main span consists of three segments, while Wilson FEM comprises one segment only. It is worth mentioning that for the 4th mode, the ordinates on the Wilson test curve at the main span are all zero. This is because no data were collected during the ambient tests due to technical issues as reported in Wilson and Liu (1991).

**Fig. 5:**
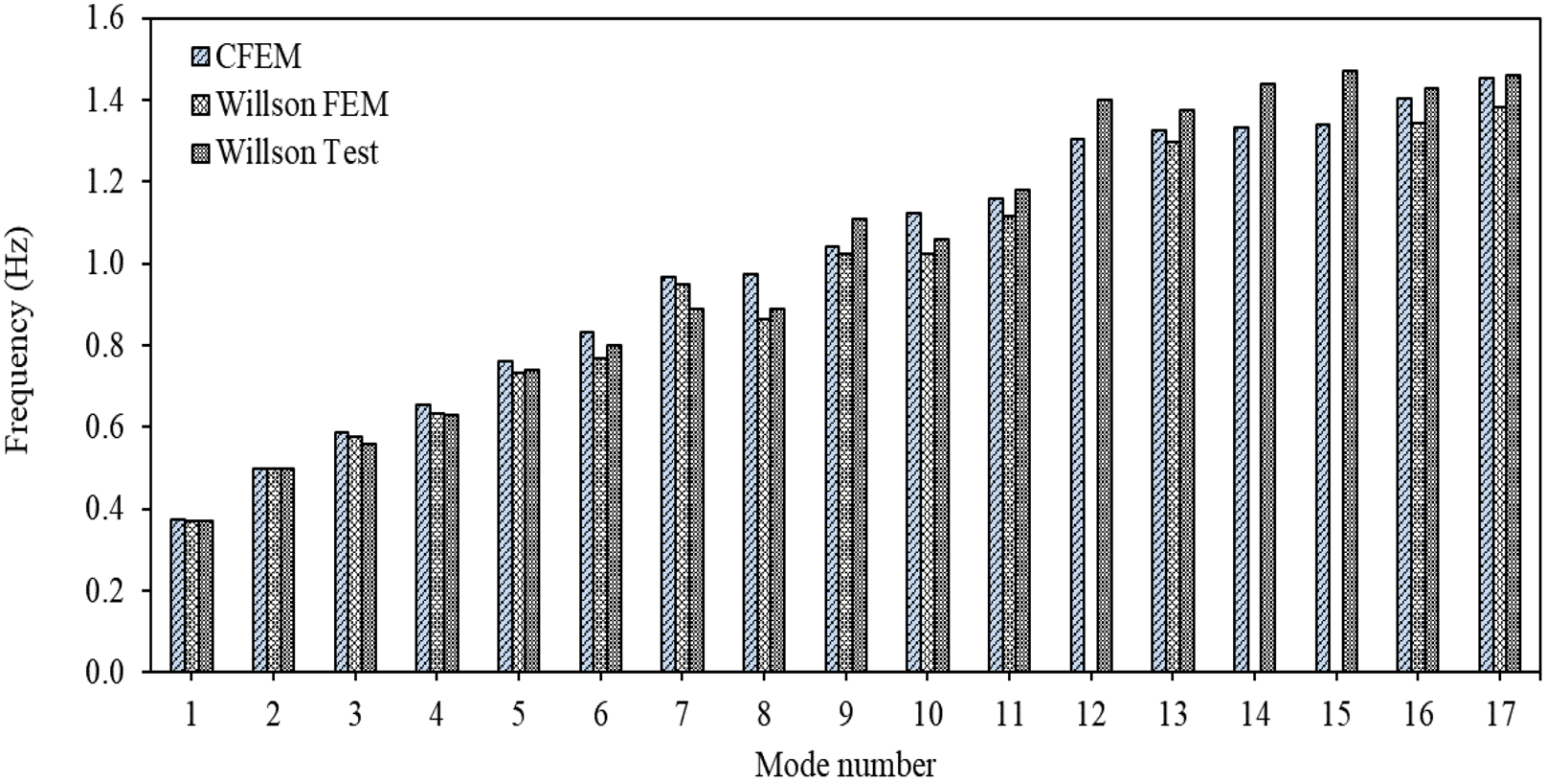
Modal frequencies from CFEM, Wilson FEM and Wilson test.

Frequency results for the first 17 modes from the CFEM, the Wilson FEM, and the Wilson test are presented in Fig. 5. It can be seen clearly in Fig. 5 that the CFEM is able to detect modes 12, 14, and 15 presented in the Wilson test, meanwhile, the Wilson FEM is unable to discover these modes. In addition, frequency results from the CFEM and the Wilson FEM are very close for the common modes detected by the two models. The maximum difference between the CFEM and Wilson test frequencies is about 0.14 Hz, which is for the 7th mode with a combined torsional-transverse movement.

Based on the foregoing discussion, it is concluded that the current finite element model (CFEM) developed is acceptable for further analysis in this study.

## Analysis Results

### Selection of Earthquake Records

For the purpose of the seismic analysis, ten records are selected from the Strong Ground Motion Database of Pacific Earthquake Engineering Research Center (PEER). Characteristics of these records are presented in Table 1. Among the ten records, eight of them are selected from earthquakes in the United States, one from the 1995 Kobe earthquake, and one from the 1999 Chi-Chi earthquake. Among the three components of each record, a time series of the horizontal component with a larger PGA is chosen as input for the time-history analysis. As given in Table 1, the PGDs of Re #1 (14 mm) from the Whittier earthquake and Re #6 (10 mm) from the Kobe earthquake are relatively small compared with other records. They are selected in this study to demonstrate that ground motions with a lower PGD would also trigger an excessive vertical deck displacement.

**Table 1:** Characteristics of the records.

Record ID	Earthquake name	Station	Mag. (M)	Dis. (km)	Comp. (Deg.)	PGA (g)	PGD (mm)	Duration (s)
Re #1	1987 Whittier	Studio City	5.99	26.91	182	0.23	14	32.39
Re #2	1984 Morgan Hill	Gilory Array # 4	6.19	11.53	360	0.34	33	39.99
Re #3	1979 Imperial Valley	Superstition Mtn Camera	6.53	24.61	135	0.20	27	28.34
Re #4	1971 San Fernando	Santa Felita Dam	6.61	24.69	172	0.15	92	39.99
Re #5	1994 Northridge	Castiac-Old Ridge Route	6.69	20.11	090	0.56	95	39.98
Re #6	1995 Kobe	Chihaya	6.90	49.91	090	0.11	10	53.99
Re #7	1989 Loma Prieta	Palo Alto-SLAC Lab	6.93	30.62	360	0.27	115	39.64
Re #8	1992 Landers	Barstow	7.28	34.86	000	0.13	146	39.98
Re #9	1952 Kern County	Taft Lincoln School	7.36	38.42	111	0.18	93	54.36
Re #10	1999 Chi-Chi	TCU095	7.62	45.15	N	0.69	255	89.99

### Preliminary Results from Synchronous and Asynchronous Excitations

**Fig. 6:**
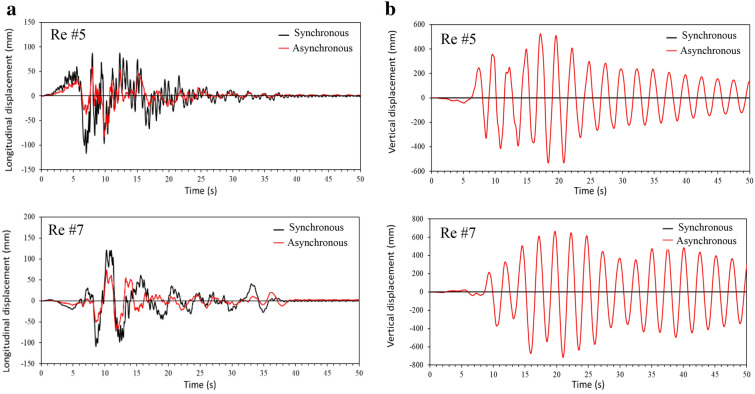
Absolute displacement time histories for point A: **a** longitudinal displacement, **b** vertical displacement.

**Fig. 7:**
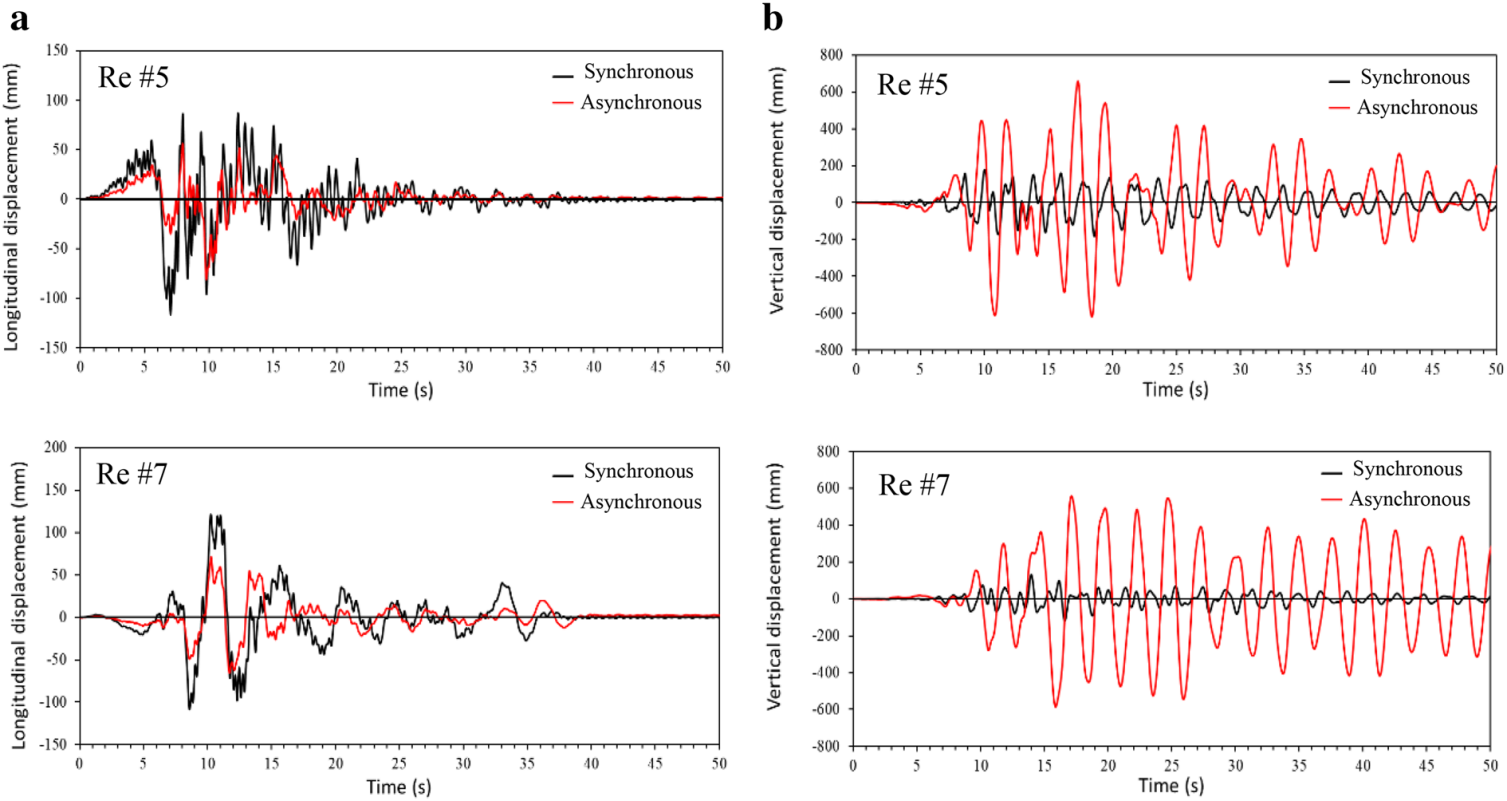
Absolute displacement time histories for point B: **a** longitudinal displacement, **b** vertical displacement.

In phase I of the study, two analysis cases are considered: one is the synchronous excitation and the other is the asynchronous excitation. For the asynchronous excitation, a seismic wave velocity of 185 m/s, which is the magnitude used in CHBDC to define Soil Class D (Stiff soil), is used to determine the time delay between the supports. In both cases, the excitation is applied to the bridge longitudinal direction for the purpose of the study, and it is assumed the wave travels from the west end to the east end of the bridge, i.e., from support 1 to support 4 (Fig. 1). The displacements at point A located at the middle of the main span and point B located at 36.6 m from point B (Fig. 1), are monitored in the analysis. Points A and B represent the locations where the maximum deck vertical displacements are observed due to synchronous excitation and asynchronous excitation, respectively. As an example, Figs. 6 and 7 present the displacements at points A and B for Record #5 and Record #7, respectively. The differences in the results from the two cases are obvious. In particular, synchronous excitation does not trigger a vertical movement at point A, while it triggers a movement at point B. By contrast, asynchronous excitation leads to excessive vertical displacement at both points. More importantly, the results in Figs. 6 and 7 show that the deck vertical displacement is about 6 times larger than the longitudinal displacement.

### Critical Seismic Wave Speed

**Fig. 8:**
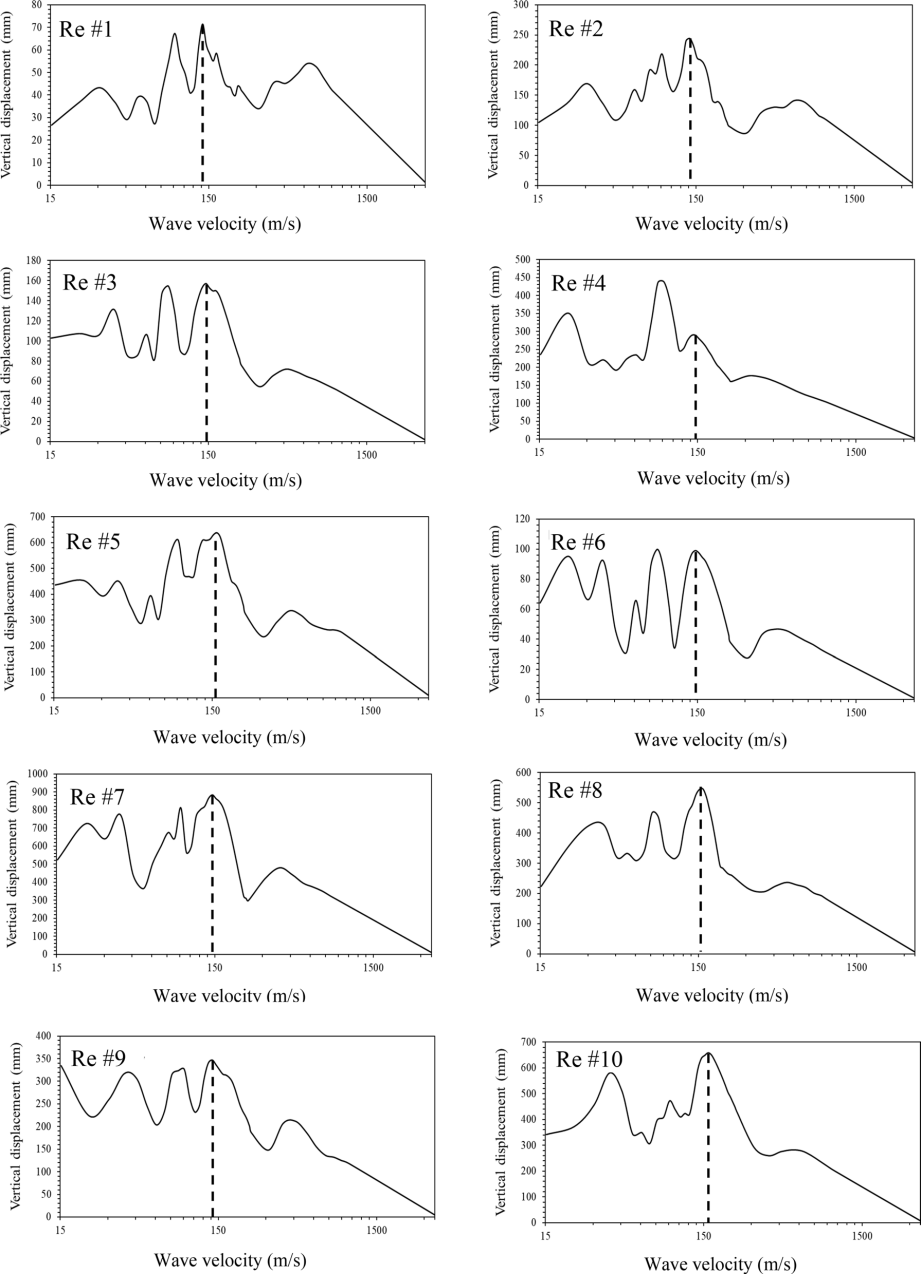
Deck vertical displacement *vs* wave velocity.

In phase II of the study, the maximum bridge deck displacement at the middle of the main span is determined by subjecting the bridge model to asynchronous excitation for each record selected. The seismic wave velocity varies from 15 to 3500 m/s at an increment of about 10 m/s. The velocity of 3500 m/s is used to represent synchronous (i.e., uniform) excitation since the delay of the wave arrival time between the piers is extremely small for the bridges under examination. Figure 8 shows the results of the deck vertical displacement at point A *vs* the wave velocity for each record. It can be seen in the figure that the peak of the displacement corresponds to a velocity of about 150 m/s, with the exception of Record #4 in which the peak occurs at a velocity of 90 m/s. As expected, for all the records, the displacement drops to zero at a velocity of 3500 m/s where the excitation is synchronous.

**Fig. 9:**
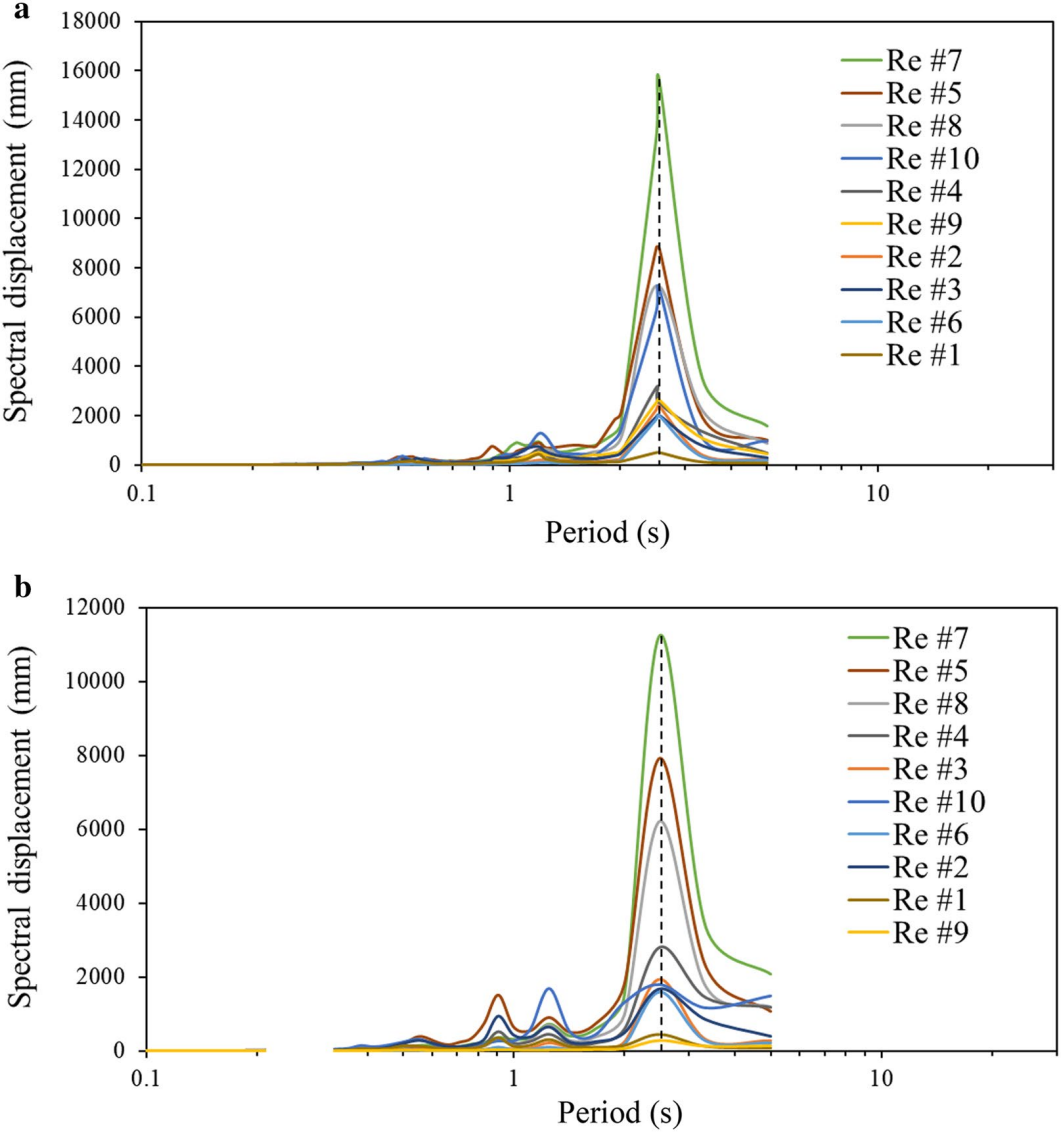
Displacement response spectra: **a** velocity of 150 m/s, **b** velocity of 90 m/s.

**Fig. 10:**
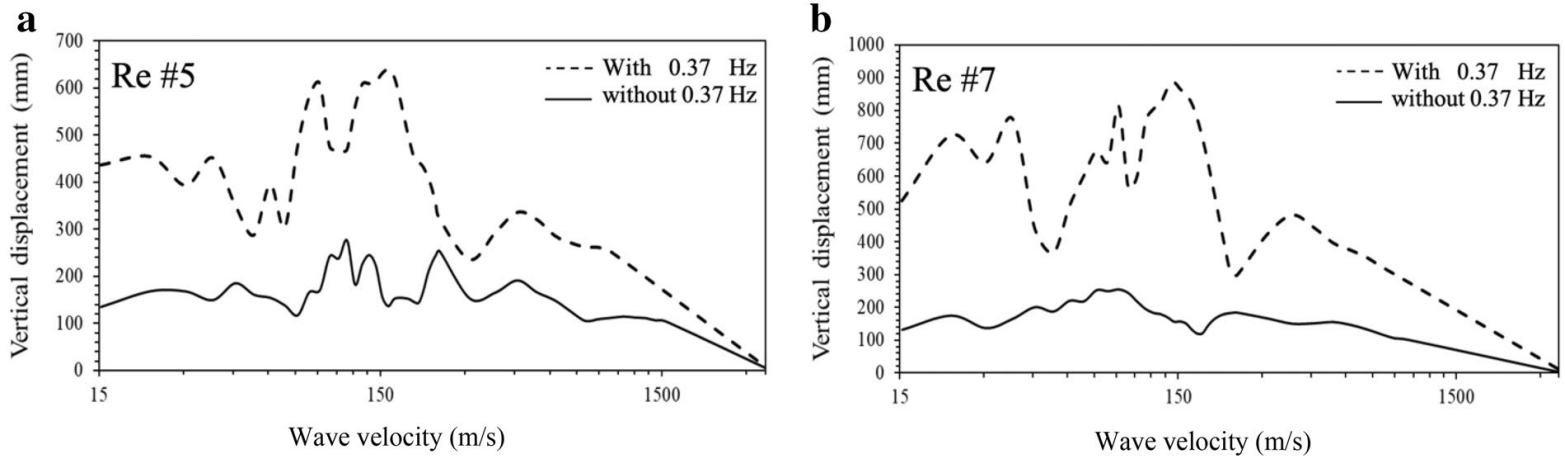
Deck vertical displacements w/o the frequency content of 0.37 Hz: **a** Record #5, **b** Record #7.

**Fig. 11:**
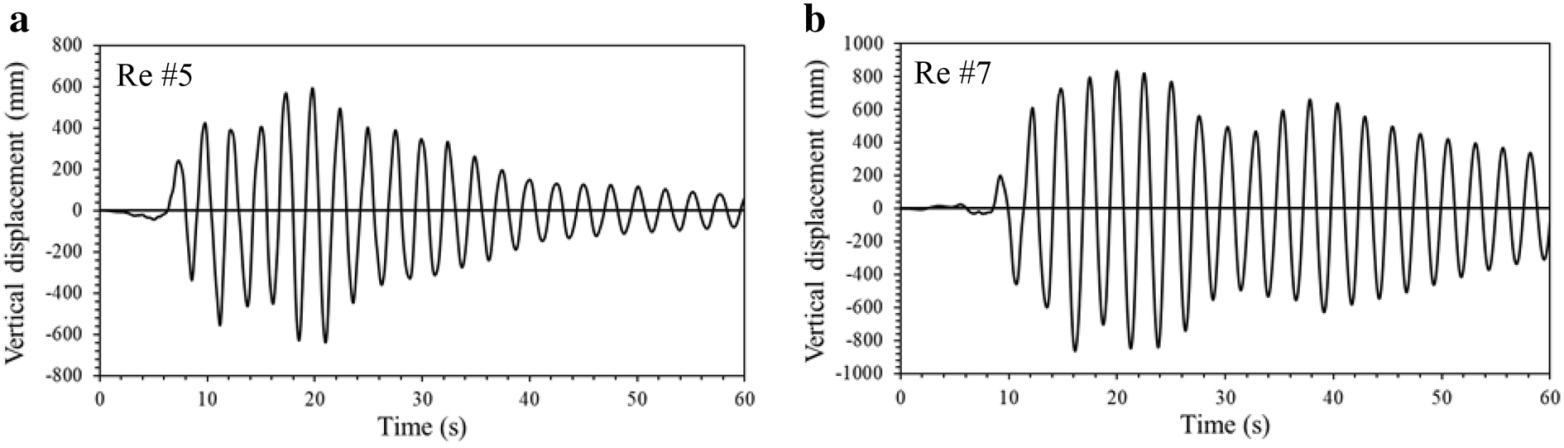
Vertical deck displacement time histories for point A: **a** Record #5, **b** Record #7.

In order to discover if higher modes contribute to deck displacement, displacement response spectra associated with the velocities of 150 m/s and 90 m/s for each record are plotted (Fig. 9). The results in the figure show that the displacement is solely dominated by the period of 2.67 s (frequency of 0.37 Hz), which is the fundamental period of the bridge model. In order to assess the effect of the frequency content of 0.37 Hz on the bridge response, this component is purposely filtered out in the original time series of each record using commercial software SeismoSignal (SeismoSoft 2018). Time-history analysis is then conducted for each "tampered" record where the frequency content of 0.37 is removed, and the maximum vertical deck displacement is collected from each run. Table 2 provides the maximum deck displacements obtained from the original records with the frequency content of 0.37 Hz and the "tampered" records without the frequency content of 0.37 Hz. The results in the table demonstrate that filtering out the frequency content of 0.37 Hz reduces the response by as much as 6 times. As an example, Fig. 10 illustrates the results of the deck displacement *vs* the velocity with and without the frequency content of 0.37 Hz in the ground motion for Record #5 and Record #7. Furthermore, it is detected that the frequency content of 0.37 Hz triggers a resonance that is reflected in the displacement time history as presented in Fig. 11.

**Table 2:** Displacement reduction ratio w/o a frequency content 0.37 Hz.

Record ID	Displacement (mm)		Ratio
	With the content of 0.37 Hz	Without the content of 0.37 Hz	
Re #1	58	26	2.2
Re #2	212	79	2.6
Re #3	152	48	3.1
Re #4	280	122	2.2
Re #5	631	156	4.0
Re #6	98	16.5	5.9
Re #7	864	157	5.5
Re #8	544	268	2.0
Re #9	325	154	2.1
Re #10	648	415	1.5

The results in Fig. 8 indicate that there is a specific wave velocity to generate the largest deck vertical displacement. This velocity is referred to as the critical velocity and designated as *V*_*c*_ hereafter. The total duration *t* of the seismic wave travelling along the entire bridge length *L* at a velocity of *V*_*c*_ can be determined using Eq. (1). In order to determine *V*_*c*_, a *C-factor* is introduced in this study as expressed in Eq. (2):

1 $$t= \frac{L}{{V}_{c}},$$

2 $$C-factor= \frac{{T}_{n}}{t},$$

where *L* = total length of the bridge; *V*_*c*_ = critical velocity to generate the maximum displacement; *t* = duration of the wave travelling along the entire bridge; and *T*_*n*_ = fundamental period of the bridge model (vertical mode).

The rationale of the *C-factor* is that once the time difference between the seismic wave arrival to the first and last supports reaches a certain fraction of the bridge fundamental period, the derived asynchronous excitation causes a resonance to the vertical deck displacement in which the response is maximized. The wave velocity associated with the excitation that produces the greatest displacement is then taken as *V*_*c*_*.* It can be seen in Eq. (1) and Eq. (2) that *V*_*c*_ depends on the bridge length *L*, the fundamental period *T*_*n*_ and the *C-factor*. Therefore, the *C-factor* needs to be quantified such that *V*_*c*_ could be estimated.

For the Bayview Bridge:

- Substitute *L* = 542 m, *V*_*c*_ = 150 m/s into Eq. (1), yield *t* = 3.61 s.
- Substitute *T*_*n*_ = 2.67 s and *t* = 3.61 s into Eq. (2), yield *C-factor* = 0.72.

### Determination of the *C-factor* Using the Proposed Equations

In order to quantify the *C-factor* proposed in Eq. (2), four additional generic cable-stayed bridges are developed based on the configuration of the Bayview Bridge. Below are the modifications made on the model of the Bayview Bridge to derive each of the new models.

Bridge #1:

- The stiffening wall between the legs of the pylon is removed.
- The vertical moment of inertia of the deck is reduced by 65%.
- The moment of inertia of pylons in the longitudinal direction is reduced by 50%.

Bridge #2:

- The vertical moment of inertia of the deck is increased four times. It should be noted that the model of the Bayview Bridge is modified in such a way that Bridge #1 has a longer period while Bridge #2 has a shorter period.

Bridge #3:

- The span length of this bridge is reduced to 393 m of which the side spans are 93 m and the main span is 201 m. These modifications are made for the following reasons: (i) the length of the main span is reduced by removing a total of eight segments from the original Bayview Bridge model; (ii) the length of the side spans is selected to keep the side-to-main span ratio of the new bridge (i.e., 0.477) as close as possible to that of the original bridge (i.e., 0.485).

Bridge #4:

- The model of this bridge is developed based on the model of Bridge #3 where the axial stiffness of its cables is increased four times and all other parameters remain the same.

Once the models of the four bridges are finalized, time-history analysis is performed on each bridge model following the same procedures as for the Bayview Bridge. Based on *V*_*c*_ and *T*_*n*_ collected from analyses of the 4 bridge models, it is found that the *C-factor* is about 0.72 as demonstrated below.

Bridge #1:

- Substitute *L* = 542 m, *V*_*c*_ = 105 m/s into Eq. (1), yield *t* = 5.16 s.
- Substitute *T*_*n*_ = 3.73 s and *t* = 5.16 s into Eq. (2), yield *C-factor* = 0.722.

Bridge #2:

- Substitute *L* = 542 m, *V*_*c*_ = 183 m/s, into Eq. (1), yield *t* = 2.96 s.
- Substitute *T*_*n*_ = 2.13 s and *t* = 2.96 s into Eq. (2), yield *C-factor* = 0.720.

Bridge #3:

- Substitute *L* = 393 m, *V*_*c*_ = 156 m/s, into Eq. (1), yield *t* = 2.52 s.
- Substitute *T*_*n*_ = 1.82 s and *t* = 2.52 s into Eq. (2), yield *C-factor* = 0.720.

Bridge #4:

- Substitute *L* = 393 m, *V*_*c*_ = 253 m/s, into Eq. (1), yield *t* = 1.55 s.
- Substitute *T*_*n*_ = 1.13 s and *t* = 1.55 s into Eq. (2), yield *C-factor* = 0.727.

### Results Validation

**Fig. 12:**
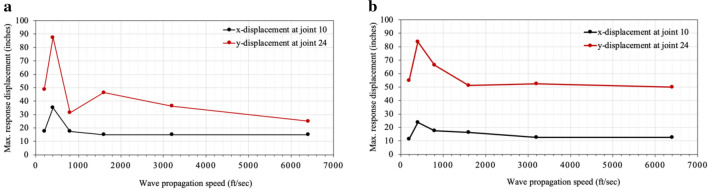
Effect of seismic wave speed on the deck displacement**: a** Model 1,** b** Model 2 adopted from Nazmy and Abdel-Ghaffar (1991).

In the study conducted by Nazmy and Abdel-Ghaffar (1991), two models were tested to examine the effects of ground motion spatial variability on the response of cable-stayed bridges. Both models have three spans: Model 1 has a center span of 1100 ft and side spans of 480 ft, while the span lengths of Model 2 are twice those of Model 1. Six wave velocities, i.e., 200, 400, 800, 1600, 3200, and 6400 ft/s, were considered in their study to derive different sets of the asynchronous excitation. Figure 12 shows the results of the deck displacement *vs* the wave velocity for the two models presented in Nazmy and Abdel-Ghaffar (1991). Joint 24 shown in Fig. 11 is located at the middle of the center span, and the y-displacement refers to the deck displacement in the vertical direction.

The critical wave velocity, *V*_*c*_, for these two bridges is determined using the *C-factor* of 0.72 and Eq. (1) and Eq. (2) proposed in this study. The calculation is as follows:

Model 1:

- Substitute *C-factor* = 0.72*, T*_*n*_ = 3.22 s into Eq. (2), yield *t* = 4.47 s.
- Substitute *t* = 4.47 s and *L* = 2060 ft into Eq. (1), yield *V*_*c*_ = 460 ft/s.

Model 2:

- Substitute *C-factor* = 0.72*, T*_*n*_ = 5.20* s* into Eq. (2), yield * t* = 7.22 s.
- Substitute *t* = 7.22 s and *L* = 4120 ft into Eq. (1), yield *V*_*c*_ = 570 ft/s.

The periods of 3.22 s for Model 1 and 5.20 s for Model 2 used in the calculation above are provided in Nazmy and Abdel-Ghaffar (1991). The calculation shows that the critical wave velocity to generate the largest vertical deck displacements in each bridge is 460 ft/s for Model 1 and 570 ft/s for Model 2. These are slightly different to the velocity of 400 ft/m observed in Fig. 12 because the velocities considered in Nazmy and Abdel-Ghaffar (1991) are discrete, and it may not therefore be possible to obtain the same maximum response given by a velocity other than with the numbers examined in their study.

## Conclusions

The objective of this study was to estimate a critical seismic wave velocity that would produce the maximum vertical deck displacement of a cable-stayed bridge. For this purpose, the Quincy Bayview Bridge located in Illinois, USA, was selected. A three-dimensional finite element model of the bridge is developed using SAP2000 and is validated with the data available in the literature. To determine the critical wave velocity, a *C-factor* and two equations are introduced in the study. The *C-factor* is a ratio of the period of the first vertical mode of the bridge model to the duration of the wave travelling along the bridge. Based on the analysis results of 5 bridges, a number of 0.72 is assigned to the *C-factor*. The *C-factor* of 0.72 is then tested on the two bridges considered in Nazmy and Abdel-Ghaffar (1991). It is found that the *C-factor* and the two equations proposed in this study predict the critical wave velocity reasonably well.

Both equations introduced in this study are straight forward and easily applied in practice. Knowing the total length of the bridge, the period of the first vertical mode of the bridge model, and taking the *C-factor* as 0.72, one can determine the critical wave velocity that will generate the maximum estimated deck displacement in the vertical direction. This critical wave velocity will help designers to quickly assess whether it is necessary to consider vertical vibration of the deck when designing, or evaluating the performance of, cable-stayed bridges under earthquake loading conditions. The results from this study also indicate that lower wave velocities tend to generate larger deck displacements in the vertical direction. It should be noted that the proposed C-factor of 0.72 is intended for use for typical 3-span cable-stayed bridges with a side-to-main ratio of about 0.48. It is equally important to note that the methodology developed in this study, however, can be applied to any specific cable-stayed bridge in order to determine whether the longitudinal seismic motion would excite the vertical deck displacement.

## Data Availability

Yes.

## References

[CR1] AASHTO. 1996. American Association of State Highway and Transportation Officials. *Standard Specification for Highway Bridges*, the 16^th^ edition.

[CR2] Abdel-Ghaffar A M 1991. Cable-stayed bridges under seismic action. Proceedings of the Seminar Cable—Stayed Bridges: Recent Development and Their Future, Yokohama, Japan.

[CR3] Allam SM, Datta TK (2004). Seismic response of a cable-stayed bridge deck under multi-component non-stationary random ground motion. Earthquake Engineering and Structural Dynamics.

[CR4] Aswathy S, Kartha U, Mathai A (2013). Seismic pounding of bridges due to multi-support excitation with traveling wave. American Journal of Engineering Research (AJER).

[CR5] Camara F (2018). Seismic behavior of cable-stayed bridges: a review. MOJ Civil Engineering.

[CR6] CEN (2004). Eurocode 8: Design of structures for Earthquake Resistance. European Standard.

[CR7] Chang KC, Mo YL, Chen CC, Lai LC, Chou CC (2004). Lessons learned from the damaged Chi-Lu Cable-Stayed Bridge. Journal of Bridge Engineering.

[CR8] CHBDC (2014). Canadian Highway Bridge Design Code.

[CR9] Clough RW and Johnston SB. Effect of stiffness degradation on earthquake ductility requirements. Transaction of Japan Earthquake Engineering Symposium, pp 195–198.

[CR10] Crewe A and Norman J. (2006). Experimental modelling of multiple support excitation of long span bridges. Proceedings of the 4^th^ international conference on earthquake engineering. Taipei, Taiwan.

[CR11] Cruz Noguez CA, Saiidi MS (2011). Shake-table studies of a four-span bridge model with advanced materials. Journal of Structural Engineering.

[CR12] De Silva CW (2005). Vibration and shock handbook.

[CR13] Filiatrault A, Tinawi R, Massicotte B (1993). Damage to cable-stayed bridge during 1988 Saguenay earthquake II: dynamic analysis. Journal of Structural Engineering.

[CR14] Fleming JF (1979). Nonlinear static analysis of cable-stayed bridge structures. Computers & Structures.

[CR15] Fleming JF, Egeseli EA (1980). Dynamic behavior of a cable-stayed bridge. Earthquake Engineering and Structural Dynamics.

[CR16] Garevski MA, Severn RT (1993). Damping and response measurement on a small-scale model of a cable-stayed bridge. Earthquake Engineering and Structural Dynamics.

[CR17] Gong Y, Park MY, Choi SH, Kim S, Cheung HJ (2015). Multi-support excitation test of single-pylon cable-stayed bridge using shaking table. Journal of the Earthquake Engineering Society of Korea.

[CR18] Guan ZG, Li JZ, Guo W, Qu HY (2019). Design and validation of a shaking -table test model on a long span cable-stayed bridge with inverted-Y-shaped towers. Engineering Structures.

[CR19] Hua, C. H., & Wang, Y. C. 1996. Three-dimensional modelling of a cable-stayed bridge for dynamic analysis. Proceedings of the 14^th^ International Modal Analysis Conference. Taiwan, pp. 1565–1571.

[CR20] Johnson N, Ranf R, Saiidi M, Sanders D, Eberhard M (2008). Seismic testing of a two-span concrete bridge. Journal of Bridge Engineering.

[CR21] Kosa, K., & Tasaki, K. 2003, Detailed investigation of PC cable-stayed bridge damage in the 1999 Taiwan earthquake. Proceedings of the 19^th^ US-Japan Bridge Engineering Workshop, Tsukuba Science City, Japan, 29–42.

[CR22] Liu, Z. J., Hong, Q. Y., Lei, H. J., & Li, X. Z. 2014. Analysis of elastic-plastic seismic response of a low-pylon cable-stayed bridge under a rare earthquake. Proceedings of the 2014 International Conference of Logistics Engineering and Management (ICLEM), Shanghai, China, October 9–11, pp. 570–574.

[CR23] Naderian H, Cheung MS, Shen ZY, Dragomirescu E (2016). Seismic analysis of long-span cable-stayed bridges by an integrated finite strip method. Journal of Bridge Engineering.

[CR24] Nazmy AS, Abdel-Ghaffar AM (1990). Three-dimensional nonlinear static analysis of cable-stayed bridges. Computers and Structures.

[CR25] Nazmy AS, Abdel-Ghaffar AM (1990). Non-linear earth quake-response analysis of long-span cable-stayed bridges: Applications. Earthquake Engineering and Structural Dynamics.

[CR26] Poddar K, Rahman T (2015). Comparative Study of Cable Stayed, Suspension and Composite Bridge. International Journal of Innovative Research in Science, Engineering and Technology.

[CR27] Pridham B, Wilson J (2005). A reassessment of dynamic characteristics of the Quincy Bayview Bridge using output-only identification techniques. Earthquake Engineering and Structural Dynamics.

[CR28] Priestley MJN, Seible F, Calvi GM (1996). Seismic design and retrofit of bridges.

[CR29] PWRI. 1998. Public Works Research Institute (PWRI). Design Specifications of Highway Bridges, Part V, Seismic Design Technical Memorandum of EED, No. 9801, Japan.

[CR30] Ren WX, Otata M (1999). Elastic-plastic seismic behavior of long span cable-stayed bridges. Journal of Bridge Engineering.

[CR31] Seismosoft (2018). Earthquake Engineering Software Solutions-SeismoSignal.

[CR32] Sextos A and Kappos A. 2005. Evaluation of the new Eurocode 8 part 2 provisions regarding asynchronous excitation of irregular bridges. Proceedings of the 4^th^ European Workshop on the Seismic Behavior of Irregular and Complex Structures, Thessaloniki, pp. 26–27.

[CR33] Shiravand MR, Parvanehro P (2019). Spatial variation of seismic ground motion effects on nonlinear responses of cable stayed bridges considering different soil types. Soil Dynamics and Earthquake Engineering.

[CR34] Siringoringo DM, Fujino Y, Namikawa K (2014). Seismic response analyses of the Yokohama Bay Cable-Stayed Bridge in the 2011 Great East Japan Earthquake. Journal of Bridge Engineering.

[CR35] Tian ZY, Lou ML (2014). Travelling wave resonance and simplified analysis method for long-span symmetrical cable-stayed bridges under seismic traveling wave excitation. Shock and Vibration.

[CR36] Wilson JC (2003). Repair of new long-span bridges damaged by the 1995 Kobe earthquake. Journal of Performance of Constructed Facilities.

[CR37] Wilson JC, Gravelle W (1991). Modelling of a cable-stayed bridge for dynamic analysis. Earthquake Engineering and Structural Dynamics.

[CR38] Wilson J, Liu T (1991). Ambient vibration measurements on a cable-stayed bridge. Earthquake Engineering and Structural Dynamics.

[CR39] Xie W, Sun LM, Lou ML (2020). Shaking table test verification of travelling wave resonance in seismic response of pile-soil-cable-stayed bridge under non-uniform sine wave excitation. Soil Dynamics and Earthquake Engineering.

[CR40] Yang CY, Cheung MMS (2011). Shake table test of cable-stayed bridge subjected to non-uniform excitation. The 12th East Asia-Pacific Conference on Structural Engineering and Construction. Procedia Engineering.

[CR41] Zadeh, O. (2012). Comparison Between Three Types of Cable Stayed Bridges Using Structural Optimization. M. A. Sc. thesis, Department of Civil and Environmental Engineering. London, Ontario: University of Western Ontario

[CR42] Zhong J, Jeon JS, Yuan WC, DesRoches R (2017). Impact of spatial variability parameters on seismic fragilities of a cable-stayed bridge subjected to differential support motions. Journal of Bridge Engineering.

[CR43] Zhou L, Wang X, Ye A (2019). Shake table test on transverse steel damper seismic system for long span cable-stayed bridges. Engineering Structures.

